# Animal Welfare in Conservation Breeding: Applications and Challenges

**DOI:** 10.3389/fvets.2018.00323

**Published:** 2018-12-18

**Authors:** Alison L. Greggor, Greg A. Vicino, Ronald R. Swaisgood, Andrea Fidgett, Deena Brenner, Matthew E. Kinney, Susan Farabaugh, Bryce Masuda, Nadine Lamberski

**Affiliations:** ^1^Department of Recovery Ecology, Institute for Conservation Research, San Diego Zoo Global, Escondido, CA, United States; ^2^San Diego Zoo Global, San Diego, CA, United States

**Keywords:** behavioral monitoring, captive breeding, conservation breeding, opportunities to thrive, welfare assessment, avian welfare

## Abstract

Animal welfare and conservation breeding have overlapping and compatible goals that are occasionally divergent. Efforts to improve enclosures, provide enriching experiences, and address behavioral and physical needs further the causes of animal welfare in all zoo settings. However, by mitigating stress, increasing behavioral competence, and enhancing reproduction, health, and survival, conservation breeding programs must also focus on preparing animals for release into the wild. Therefore, conservation breeding facilities must strike a balance of promoting high welfare, while minimizing the effects of captivity to increase population sustainability. As part of the Hawaii Endangered Bird Conservation Program, San Diego Zoo Global operates two captive breeding facilities that house a number of endangered Hawaiian bird species. At our facilities we aim to increase captive animal welfare through husbandry, nutrition, behavior-based enrichment, and integrated veterinary practices. These efforts help foster a captive environment that promotes the development of species-typical behaviors. By using the “Opportunities to Thrive” guiding principles, we outline an outcome-based welfare strategy, and detail some of the related management inputs, such as transitioning to parental rearing, and conducting veterinary exams remotely. Throughout we highlight our evidence-based approach for evaluating our practices, by monitoring welfare and the effectiveness of our inputs. Additionally we focus on some of the unique challenges associated with improving welfare in conservation breeding facilitates and outline concrete future steps for improving and evaluating welfare outcomes that also meet conservation goals.

## Introduction

A good state of welfare is generally representative of animals that are well nourished, safe, lack pain, fear, and distress, and have the ability to develop and express species-typical relationships, behaviors, and cognitive abilities (1–3). Measuring and accomplishing these aims requires tailored approaches, since the needs of every species (and individual) can be different. Moreover, an animal's welfare state can change temporally, with development, or with fluctuating external stressors (2). Therefore, welfare goals need to be assessed with regularity, even when management actions have not changed.

At San Diego Zoo Global (SDZG), we address the necessity and complexity of meeting the needs of species and individual animals through our Opportunities to Thrive program (Table 1). Developed to replace the seminal Five Freedoms established by the UK's Farm Animal Welfare Council (4), this program provides guidance for managing all animals in our collection, and our conservation breeding programs. Conservation breeding involves the captive propagation of endangered species to help maintain genetic diversity, produce viable individuals for release and ultimately mitigate species' extinction (5). Positive indicators of animal welfare are essential components of effective conservation breeding programs because they are correlated with reductions of physiological indicators of stress (6), the incidence of health issues (7), and increases in reproductive success (8). However, unlike many zoo settings, conservation breeding facilities need to execute a welfare strategy explicitly aimed at increasing the likelihood of successful reintroductions to the wild.

**Table 1:** Summary of welfare actions, organized by the Five Freedoms and their relation to the Opportunities to Thrive.

**Freedom**	**Opportunity**	**Management actions (inputs)**	**Assessment techniques (outputs/outcomes)**
From hunger and thirst	Strategically presented, well-balanced diet	Formulate diets to meet species' requirements through life history, including breeding, and chick rearing	Records kept for food consumption and food type preferences, measurements of body scores, and weight that assess pectoral muscle condition and fat stores
From discomfort	Self-maintain	Aviaries designed for shelter, adequate perching, room for flight, with minimal human contact	Behavioral observations of stress and positive self-maintenance behavior
From pain injury or disease	Optimal health	Telemedicine health checks, animal care staff training	Documentation of health based on physical exam, body weight, and condition, and biomaterial evaluation (blood, feces, tissue)
To express normal behavior	Expression of species-typical behavior	Offer native foods, naturalistic enrichment, nest building, pair bonding, parental care	Behavioral observations of pair bonding, nest activity. Appropriate use of enrichment.
From fear and distress	Choice and control	Dynamic perching, social housing options, multiple nest platforms, and opportunities for mate choice	Using behavioral observations to measure the choices made and amount of time engaged with options presented

*These opportunities differ from the five freedoms by focusing on positive indicators of welfare, rather than the absence of negative ones*.

One SDZG program that has embraced the Opportunities to Thrive in pursuit of conservation breeding goals is the Hawaii Endangered Bird Conservation Program (HEBCP). The HEBCP seeks to prevent extinction and support the recovery of wild populations, primarily using captive propagation, and reintroduction techniques, alongside state, federal, and local partners. We have cared for a total of 16 endemic Hawaiian species across our two breeding facilities, including most recently, Nene (*Branta sandvicensis*; Hawaiian goose), Puaiohi (*Myadestes palmeri*), Palila (*Loxioides bailleui*), ‘Alalā (*Corvus haweaiiensis*), and ‘Akikiki (*Oreomystis bairdi*). Some species recovered sufficiently for us to end their conservation breeding programs (e.g., Nene and Puaiohi), and others have been recently added as their conservation status has declined in the wild (e.g., ‘Akikiki). Since 1993, we released over 800 Hawaiian birds into the wild.

The Opportunities to Thrive guides our integrated animal management strategy by lending structure to our welfare efforts. We use each opportunity to highlight a set of desired outputs (e.g., increased foraging or fewer stress-related behaviors), which we target with a series of inputs (i.e., welfare-focused management actions). We then evaluate whether our actions solicit the intended outputs, which allows us to better plan future inputs. There is overlap between tactics for addressing the opportunities, so these should be viewed as a broad coordinated approach. We summarize our methods in Table 1, and describe our rationale and the challenges we face throughout the paper. While some details to our approaches may be unique to the species under our care, the application of these principles are not limited to avian facilities.

## Opportunity for a Strategically-Presented, Well-Balanced Diet

Avian species show considerable variation in their natural diets and welfare of birds in captivity depends heavily on meeting their nutritional needs through normal foraging and feeding behavior. Diets should provide all necessary nutrients, and be of adequate quantity, quality, and variety. Food also needs to be presented in a manner and a frequency appropriate to the species, in a way that can facilitate an evaluation of dietary choices. The individual animal's condition, size, physiological, reproductive, and health status must also be considered during diet formulation (9). Imbalanced diets can be linked to poor health (e.g., (10, 11) with associated veterinary costs, poor egg production, and low chick viability (12).

Even if an optimal diet is offered, it cannot be assumed that animals consume the desired proportion and quantities of its components. We use common hands-on evaluation techniques, such as weighing feed intake when hand-rearing chicks by placing them on a scale during feeding sessions, and calculating the nutrient composition of items consumed (10, 13). Meanwhile, for our birds that require space and privacy to rear their own young, we assess these measures observationally. We monitor parental interactions at the nest via CC-TV, noting how many times each parent feeds their chick during a set period of the day, and we record the quantities and type of food that parents have removed from their food pan when it is collected at the end of the day.

On the infrequent occasion that we handle a bird, we conduct assessments of their body condition, scoring their muscle mass, and fat reserves in addition to taking weight. Body condition scoring (BCS) is a numerical, subjective measurement of muscle definition, and superficial fatty tissue, which helps assess a bird's general health relative to their food supply. Low BCS scores are associated with lowered reproductive success, poor recovery from illness, and with disease or age (14). High BCS scores are associated with reproductive disorders, arthritis, diabetes, and other chronic conditions (15). Although BCS is an effective tool, the scoring system used for each species can be different. For instance, we adapted a pectoral muscle and fat store scoring system (16) for ‘Alalā and Palila.

Optimizing nutrition in a captive breeding setting can be a challenge without data on the quantities wild birds eat, or on the chemical, and nutritional composition of native foods. Moreover, since the provision of food offers opportunities for animals to display natural feeding behaviors, an understanding of species-typical foraging, and food processing is required to assess desired nutritional welfare outcomes. For these reasons, we evaluate not just the nutritional aspects of feeding, but also the foraging competency of our birds. For instance, wild Palila forage primarily by prying open seed pods from a native tree; a foraging skill that captive birds can lack (17). We provision the birds with a predetermined number of pods and track their foraging proficiency by later checking how many remain, and how many pods are opened successfully.

## Opportunity to Self-Maintain

Animals need the opportunity to engage in positive behaviors to proactively avoid discomfort and rest when appropriate. Examples of these behaviors include self-grooming and bathing, as well as the ability to move freely and avoid undesirable weather or social conditions. Self-maintenance behaviors are a common positive welfare marker (18).

We designed our aviaries with the opportunity to self-maintain in mind. While the exact dimensions of the aviaries vary by species, each bird is provided with ample areas to shelter in native vegetation, roost, bathe, fly, feed, perch, and walk, all while minimizing human contact. To evaluate whether these inputs actually promote self-maintenance, our team conducts twice daily health and well-being checks, often from behind one-way glass. The team also meets daily to discuss observations of unusual behaviors that may be cause for concern or be markers of improvement. These daily observations and discussions are distilled into a written daily report that is circulated to all relevant off-site staff, such as veterinarians. If an issue arises, immediate monitoring is initiated, but for chronic issues, a longer term behavioral assessment protocol is enacted. For instance, after keepers voiced concerns that daily husbandry activities (e.g., feeding and cleaning) could increase stress-related behaviors, we devised an observational protocol to determine if housing or husbandry inputs could alter stress outcomes. These observations assessed self-maintenance (e.g., preening) and stereotypic or stress behavior (e.g., pace-flying) before and after routine husbandry to best measure changes to welfare outcomes during daily routines.

Conservation breeding environments necessitate limited human-animal contact in order to ensure the birds are as wild as possible in preparation for future releases. However, this limits the usage of hands-on training for welfare checks, and increases our reliance on remote monitoring, such as CC-TV or hidden observation areas, to track positive indicators of welfare.

## Opportunity for Optimal Health

We strive for more than the absence of pain and disease, and instead aim to foster healthy well-being. This shift means we proactively look for positive markers of health, instead of waiting for the negative consequences of poor health to manifest.

A team of SDZG veterinarians and registered veterinary technicians provide on-site medical care at both breeding facilities and ‘Alalā release facilities 2–3 times per year. During on-site visits, birds are examined to follow up on existing medical issues or to diagnose and treat new medical concerns. This may include physical or visual examinations, diagnostic imaging, triage care, surgery, and biological sample collection and analysis. Birds in the release program are examined to ensure fitness and health prior to release. Biological samples are collected during the pre-release exam process and also from birds in the conservation breeding program for future disease investigations. Capacity building with staff during onsite visits fosters collaboration and allows opportunity to train staff in essential skills.

Due to the remote nature of this conservation effort, immediate on-site medical care by a veterinarian is not always possible. As a result, HEBCP husbandry staff has been trained by SDZG veterinary staff in basic treatment and diagnostic sample collection techniques. This onsite training provides a platform to efficiently practice telemedicine (e.g., practicing remote, electronically communicated health care) through video conferencing, photograph review, and phone consultations (19). Diagnostic blood samples, fecal samples, and carcasses are processed on-site by HEBCP staff and sent by overnight mail to SDZG for evaluation by SDZG pathologists and clinical veterinarians. This turn-around allows for rapid evaluation of samples and response to medical cases.

Providing veterinary care at remote sites provides unique challenges, but the close collaboration with HEBCP husbandry and field staff, SDZG clinical veterinarians, and veterinary pathologists, nutrition, and laboratory staff helps us provide the highest quality of care in this conservation breeding program.

## Opportunity to Express Species-Typical Behavior

While it has long been recognized that the performance of species-typical behavior can have positive outcomes for animal welfare, the concept has been unevenly applied across zoo settings (20). Sometimes referred to as “ethological needs” or “behavioral needs” (21), there is growing evidence that animal welfare is improved by the performance of species-typical behaviors. An environmental enrichment program that addresses these behavioral needs can reduce stress and stereotypic behavior (20, 22). In addition to the welfare benefit, maintaining natural behaviors in conservation breeding programs is important because artificial captive environments can prevent the development of survival skills, such as foraging, escaping from predators, and navigating unknown spaces (23).

In addition to supporting natural feeding and self-maintenance behavior with targeted welfare inputs, we also encourage the expression of normal social behavior; an indicator of positive animal welfare (24). We address this by housing birds in species-typical social arrangements. For example, young ‘Alalā are gregarious in the wild, but adults are territorial. Therefore, we house them in age cohorts comprised of 4–6 individuals until they reach maturity, and then transition them to single-pair breeding aviaries separated by at least 100 meters. We have preliminary data suggesting that ‘Alalā pairs may have greater reproductive success in distant compared to proximate aviaries, indicating that the welfare benefits associated with greater privacy from other pairs may positively influence reproduction.

Beyond the social setting, we designed inputs to allow the expression of species-typical breeding behavior. Nest-building, egg incubation, chick rearing, and other behaviors associated with the reproductive cycle are critical behavioral needs. These behaviors can be all-consuming, and divert birds' attention away from expression of problem behaviors such as stereotypies. Thus, we offer potential breeding pairs a variety of nesting material, allowing them to construct their own nests. Not only does this provide an enrichment opportunity, but by gauging the level of interest and investment in nest building behavior, our team can determine the breeding phase of birds. For instance, ‘Alalā engage in a “cup form” behavior in the later stage of nest building, and a peak in this behavior indicates that the female is likely to lay her first egg of a clutch (25). This behavioral outcome is critical for predicting when to use adaptive management strategies to increase the likelihood of a successful hatch.

Due to the incredible value of each egg and chick, the early stages of many conservation breeding programs focus on the survival of chicks to retain the genetic diversity of target species. However, as captive flocks grow and species-specific rearing techniques improve, there is often more capacity for the expansion of parental rearing. Accordingly, HEBCP has a long history of conducting highly skillful artificial incubation and chick rearing, and over time we shifted away from artificial rearing and to allow more birds to incubate and provide care for their own offspring. Additionally we are moving toward allowing mate choice for new pairs (see next section). These husbandry changes no doubt address a suite of pair-bonding and parental behaviors whose performance is beneficial for the welfare of the birds because they encourage species-typical behaviors, and alleviate the potential negative welfare outcomes associated with “forced” mate pairing (26) and removal of offspring (27).

We also manage a larger experience-based enrichment program that includes the provision of additional opportunities to perform species-typical behavior, such as problem solving, so that lessons learned can guide future enrichment strategies. Monitoring the outcomes of these provisions, such as individual-level engagement with provided enrichment, allows us to continually adapt and increase challenges as birds reach release. Through our iterative process of observing, learning, and managing the birds in our care, we continually improve our approximations of their wild-type behaviors, which are otherwise poorly understood.

## Opportunity for Choice and Control

Having choices allows animals to exert control over their environment, which helps regulate emotional responses to stressful situations (28). In zoo settings, having choices about space use, or social interactions can reduce behavioral and hormonal signs of stress (29, 30). We give choices to the birds in their everyday lives. Each aviary has numerous perch types that vary in height, and level of cover. We offer breeding pairs multiple aviary chambers, so they can chose their proximity to their mate. Particularly in breeding season, we evaluate each pair's amount and type of social contact with behavioral observations, and take action to separate or resocialize birds depending on pair interactions (see Table 2 for example of observation protocols). Recently we also began giving birds a choice in where to build their nest and the amount of supportive infrastructure provided, allowing them artificial, but “easy,” or natural, but “more difficult” opportunities. Implementing these nesting options served a dual purpose because we measured preferences for a given nest type, while also offering the birds an additional choice.

**Table 2:** Example observation protocol: ethogram for monitoring ‘alalā breeding pair interactions.

**Behavior**	**Definition**
**OCCURS IN 2 MINUTE PERIOD?**	
Proximity	The birds come within one body length of each other for at least 10 s.
Co-attention	While foraging or searching for food or sticks both birds focus on the same item or area. Both beaks must be oriented toward the same item or area, close enough to allow potential aggression. Examples include both birds inspecting the same crevice or both pulling food from the same food item.
Contact sitting	Two birds sit touching sides or within one body width apart (not jumping around or feeding). Birds may twine necks around each other.
Allopreening	One bird preens, nibbles, or rubs another with head, beak, or neck; if mutual, score behavior for both birds involved, e.g., birds A and B are preening each other at the same time, score A:B and B:A.^*^
Beg	Bird pumps head up and down while holding wings out and pumping them up and down. Can be accompanied by a begging call.
At nest	Bird stands or sits on one of the nest types (crown or tub) for at least 5 s. Please mark the bird and type of nest.
Nest build	Bird interacts with a stick, grass, or other nest material while standing on either the nest tub or crown. Please mark the bird and type of nest (crown or tub).
**OCCURS IN 30 MINUTE PERIOD?**	
Threaten	Threatening behavior that does not involve physical contact toward another bird. Includes: raising scapular feathers, head down threat, head up threat, lunges, attempts at biting, pecking, or striking with wing, foot, etc., flight buzz, successful and unsuccessful attempts to steal an object or food item (without contact being made)^*^
Contact aggression	One bird aggresses another and makes actual physical contact. Examples include: biting, pecking, striking, or landing on another bird, moving another bird's head away with the aggressor's own head/beak, successful, and unsuccessful attempts to steal an object or food item (with contact being made).
Pace fly	Bird flies rapidly back and forth along the length of the aviary. Each pass (one length of the aviary) counts as one pace.
Cup form	Bird lays on their stomach in the nest (almost like a belly flop) and wriggles feet/wings. A pause in the wriggling motion denotes the end of one cup form. Multiple cup forms can occur in short periods of time and each should be noted.
Cooing	Typically a male behavior. The male makes a cooing noise while dipping his head up and down below his shoulders.
Copulate	Two birds attempt or succeed in copulating. Copulations are characterized by the female tail wagging and the male mounting the female.

*Thirty minute observations using this guide aim to capture aggression, pair bonding, and measure stress. The 30 min are broken into 2 min time chunks. Relatively common behaviors are noted only once per 2 min period if they occur, and rarer behaviors are noted every time they occur in the 30 min period. If birds express threats, contact aggression or pace flying, it indicates lower levels of pair compatibility, and higher levels of stress. In contrast, pairs that exhibit relatively more allopreening, allofeeding, and contact sitting are considered to be well bonded and exhibiting positive signs of welfare*.

* *behaviors adapted from Jolles et al. (31)*.

Having choice can also matter when it comes to picking a mate. In giant pandas, for example, allowing free mate choice before pairing can improve the reproductive success of pairs, especially if the choice is mutual between both members of the pair (32). However, metrics for mate choice can differ by species, and there is a dearth of information on the breeding behavior of many endangered species. Therefore, when establishing a new breeding population with ‘Akikiki, we immediately set up opportunities for mate choice and behavioral observation protocols to help us explore how to monitor breeding preferences by systematically measuring stress and pair bonding behaviors. As a result of this effort, ‘Akikiki bred for the first time in captivity.

## Conclusions

Despite the successes of HEPCP, there is still much to learn about optimizing welfare and the breeding potential for each species. While we consider high standards of welfare to be a priority goal for all species, we identified several challenges that are applicable to the care of many endangered species. For instance, the lack of knowledge about species-typical diets and behavior can make assessments difficult. Additionally, the need to stay as “hands-off” as possible means we cannot rely on traditional operant training techniques and instead must utilize behavioral observation in multiple contexts. By continuing our efforts to research and monitor the birds, we hope to continue improving our welfare outcomes alongside our conservation goals. The more we learn about the unique species under our care, the more we can provide them with opportunities to thrive.

## Author Contributions

AG, RS, and NL contributed conception and design of this manuscript. AG wrote the first draft of the manuscript. NL, GV, RS, MK, AF, DB, SF, and BM wrote sections of the manuscript. All authors contributed to manuscript revision, read and approved the submitted version.

### Conflict of Interest Statement

The authors declare that the research was conducted in the absence of any commercial or financial relationships that could be construed as a potential conflict of interest. The reviewer ESH declared a shared affiliation, with no academic collaboration, with one of the authors GV to the handling editor at the time of review.
